# Strong association between cervical and breast cancer screening behaviour among Danish women; A register-based cohort study

**DOI:** 10.1016/j.pmedr.2018.10.017

**Published:** 2018-10-27

**Authors:** S.H. Larsen, L.F. Virgilsen, B.K. Kristiansen, B. Andersen, P. Vedsted

**Affiliations:** a Research Unit for General Practice, The Research Centre for Cancer Diagnosis in Primary Care (CaP), Bartholins Allé 2, 8000 Aarhus C, Denmark; b Department for Public Health Programs, Randers Regional Hospital, Skovlyvej 1, 8930 Randers, Denmark; c Department of Clinical Medicine, Aarhus University, Denmark

**Keywords:** Cervical cancer screening, Smear, Breast cancer screening, Mammography, Non-participation, Coverage, Denmark

## Abstract

High coverage is essential for the effectiveness of national screening programmes. Identifying non-screeners across different screening programmes may help inform strategies to improve uptake. This study aims to analyse the association between previous cervical cancer screening (CCS) coverage and participation in breast cancer screening (BCS). This historical register-based cohort study included 91,787 Danish women aged 50–64 years who were invited to participate in the first organised round of BCS in the Central Denmark Region (CDR) in 2008–09. CCS coverage was defined as having a smear registered in the 5 1/2 years preceding the BCS, and BCS participants were divided into participants and non-participants and further categorised as active non-participants (ANP) if they cancelled and passive non-participants (PNP) if they abstained from the appointment. Of all 91,787 women included in the study, 62,391 (68%) were covered both by CCS and participated in BCS. Women not covered by CCS were more likely to be non-participants in BCS than women covered by CCS (PRRadjusted = 2.80, 95% CI: 2.68–2.93). Both PNP (PRRadjusted = 3.99, 95% CI: 3.80–4.19) and ANP (PRRadjusted = 2.50, 95% CI: 2.34–2.68) were more likely not to be covered by the CCS. In conclusion, non-coverage by CCS was strongly associated with nonparticipation in BCS. Specific groups of women only participated in one screening programme. To increase uptake, future interventions may specifically target these groups.

## Highlights

• One in ten did not participate in neither cervical nor breast cancer screening.
• Two thirds of the 91,787 included women participated in both screening programmes.
• Non-participation was strongly associated across the two screening programmes.
• Cervical non-screeners tended not to cancel when abstaining from breast screening.

## 1 Introduction

Cancer is one of the most common causes of death in Western countries including Denmark (Naghavi et al., 2015) and therefore represents a serious burden to society and challenge to the healthcare system. Early diagnosis and timely treatment are associated with better prognosis. Many countries have therefore introduced organised cancer screening programmes, e.g. cervical cancer screening (CCS) and breast cancer screening (BCS). Screening is associated with a reduced incidence and mortality of cervical cancer (CC) and in combination with improved treatment (Welch et al., 2016), screening has been shown to reduce mortality from breast cancer (BC) (Klint et al., 2010; Independent UK Panel on Breast Cancer Screening, 2012).

In Denmark, local screening for CC began in the 1960s, and was gradually rolled out nationally (Lynge et al., 2017), whereas BCS began locally in the 1990s and was implemented nationally in 2008 (Vejborg et al., 2011). High coverage is pivotal to achieving screening programme effectiveness; yet, far from all women participate in the programmes. In 2016, the participation rates were 64% for CCS and 82% for BCS in Denmark (Danish Quality Database for Cervical Cancer Screening, 2016; Danish Quality Database for Mammography Screening, 2016). Differences in participation in CCS and BCS may be due to age differences in the target groups, differences in the examination and sample collection, in invitations and in knowledge about the cancer types. Non-participation may be associated among others with linguistic or cultural barriers (Leinonen et al., 2017; Jensen et al., 2012; Harder et al., 2018), psychosocial barriers (Harder et al., 2018; Lagerlund et al., 2015), lack of resources (Leinonen et al., 2017; Jensen et al., 2012; Harder et al., 2018; Lagerlund et al., 2015) and fear of side effects (Glasgow et al., 2000).

Previous studies have reported an association between participation in CCS and BCS (Miedema and Tatemichi, 2003; Labeit and Peinemann, 2015; Augustson et al., 2003; Rakowski et al., 1995; Schoofs et al., 2017; Blackwell et al., 2008; Cummings et al., 2000; Sutton et al., 1994; Aro et al., 1999; Phillips et al., 1998). However, these studies were based on self-selection and self-reported data in which overestimation of screening participation has been reported (Howard et al., 2009). To our knowledge, no studies have used solely register-based data to examine the association between non-participation in screening for CC and BC. Therefore, the aim of this study was to analyse the association between CCS coverage and participation in BCS.

## 2 Methods

### 2.1 Setting and study design

A historical register-based cohort study including women invited for the first organised screening round for BC was conducted.

In Denmark, screening programmes, as well as health care in general, are tax-funded and all residents have free access to health care (Ministry of Health, 2016). The first screening round for BC was implemented in the Central Denmark Region (CDR) during 2008–2009, where all women aged 50–69 years received an invitation with a specific, but changeable booking date for BCS (Vejborg et al., 2011). Non-participants received no reminders in the first screening round.

Screening for CC has been offered nationally every third year to all Danish women aged 23–59 years since 2006. From 2007 onwards, the screening interval was extended to include women aged 23–64 years, and for 50–64-year-old women the screening interval was changed from every third to every fifth year (Danish Health Authority, 2012). Prior to 2006, screening was organised by 16 counties in Denmark, which caused small variations in the organisation of screening. However, 23–59-year-old women living in the current CDR have been offered BCS since 1996. CCS is performed by a general practitioner (GP) or a gynaecologist who obtain cervical smears for analysis. Smears are also used in cases where women are followed up for previous cervical intraepithelial neoplasia. Women are invited for screening by postal mail if they have not had a smear in the preceding 3 or 5 years (depending on age), and they must subsequently book an appointment with their GP to participate in the programme. In case of absence, reminders are sent to the woman, the first after 3 months, the second after 6 months.

All persons living in Denmark have a unique 10-digit identification number, called the civil personal registration number (CPR). This number is registered at every contact with public authorities, including the healthcare system (Pedersen, 2011), and the CPR enables linkage of register data at the level of the individual.

### 2.2 Study population

**Fig. 1:**
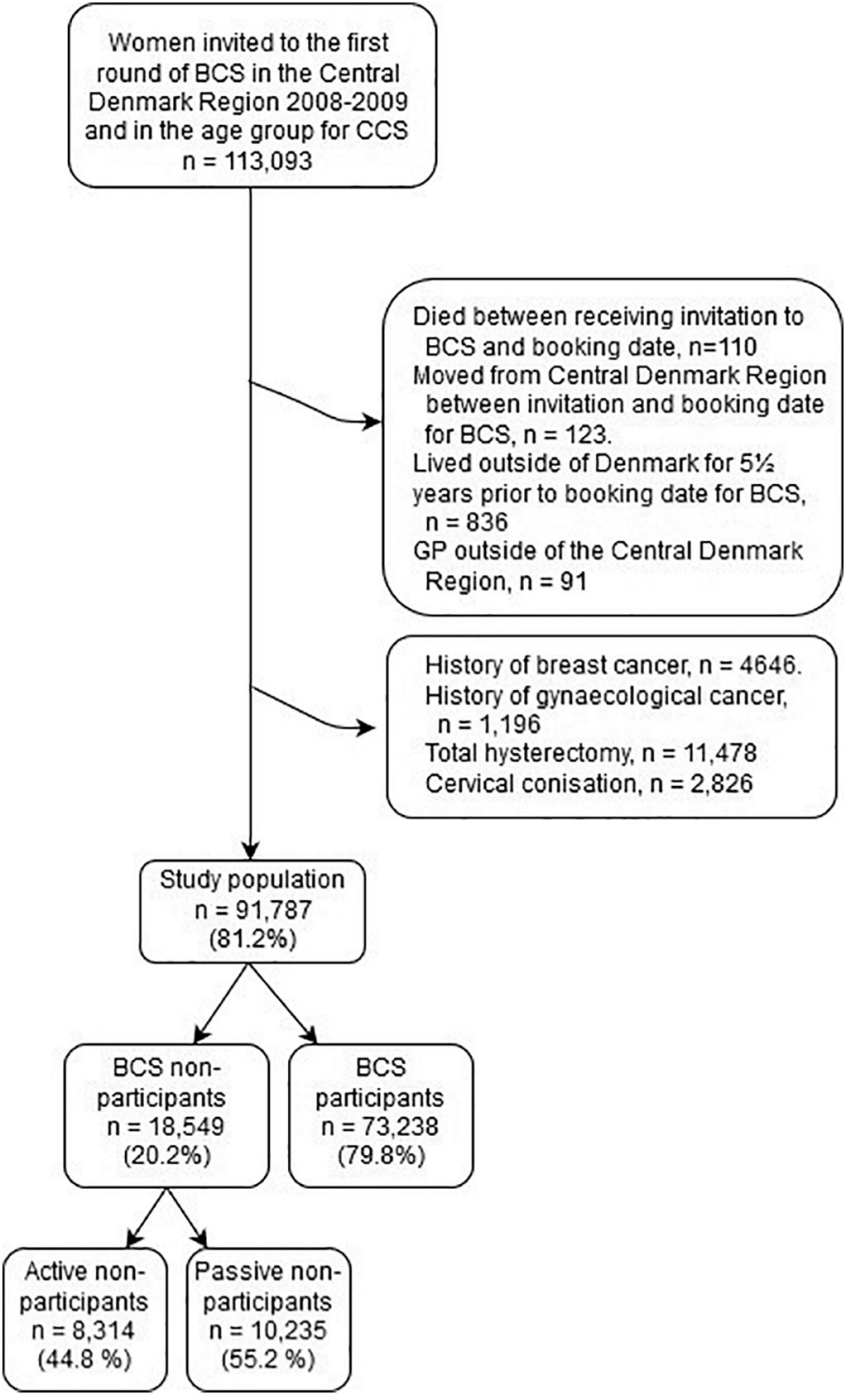
Study flowchart for women invited to the first round of breast cancer screening (BCS) in the Central Denmark Region (CDR) in 2008–2009 within the relevant age group for cervical cancer screening (CCS).

The study cohort consisted of all women aged ≤ 64 years invited to the first screening round for BC in the CDR in 2008–2009 (*n* = 113,093). Excluded were women (Fig. 1) who had died (*n* = 110) or moved away from the CDR (*n* = 123) between receiving the invitation for BCS and the booking date, women who had lived outside of Denmark for 5 1/2 years prior to the booking date for BCS (*n* = 836), and women who were registered with a GP outside of the CDR (*n* = 91). We also excluded women if they were registered with a previous history of BC (*n* = 4646) (registered with ICD-10 code C50 in the Danish Cancer Register (DCR) (Gjerstorff, 2011)) or gynaecological cancer (*n* = 1196) (registered with ICD-codes C51-C58 in the DCR). Furthermore, women registered with previous cervical conisation (*n* = 2826) were excluded (procedure codes: KLDC00 and KLDC03 in the National Patient Register (NPR) (Lynge et al., 2011) or SnoMed-code: T83701 in the National Pathology Data Bank (NPDB) (Bjerregaard and Larsen, 2011)). Finally, we excluded women who were registered with previous total hysterectomy (n = 11,478), identified using the procedure codes in the NPR: KLCD00, KLCD01, KLCD04, KLCD10, KLCD11, KLCD30, KLCD31, KLCD40, KLCD96, KLCD97, KLDC13, KLDC20, KLDC23, KLEF00B, KLEF13 and KMCA33, or SnoMed-codes in the NPDB: T81900, T82000, T82900, T82920 and ÆAA030.

In total, the study included 91,787 (81.2%) women invited to the first organised round of BCS within the relevant age group for CCS in the CDR.

### 2.3 Data collection and variables

The main outcome was participation in the first BCS round in the CDR. Data on participation in the BCS were collected from an administrative database. Women were divided into participants and non-participants in BCS. As previously described by Jensen et al. (Jensen et al., 2012), non-participants were subdivided into *active non*-*participants* (ANP) who cancelled their appointment and *passive non*-*participants* (PNP) who did not show up on the booking date.

CCS coverage was defined as having a minimum of one smear registered in the NPDB in the preceding 5 1/2 years before the BCS booking date. Women with a smear registered prior to this time or no smear at all were defined as not covered by CCS. The NPDB has existed since 1999 and every pathological specimen taken in Denmark is registered in the NPDB (Bjerregaard and Larsen, 2011). The time interval of 5 1/2 years was chosen to ensure that all women, including those entitled to screening every five years, had received at least one CCS invitation and reminders if relevant during the study period.

Information on confounders was obtained from Statistics Denmark. *Age* was included as a continuous variable. *Educational* level was categorised according to the UNESCO classification (UNESCO Institute for Statistics, 2012) as ‘low’ (≤10 years), ‘intermediate’ (11–15 years) and ‘high’ (>15 years). *Ethnicity* was categorised as ‘Danish/descendants’ and ‘immigrants’; and *marital status* into ‘married’, ‘cohabiting’ and ‘living alone’. *Income* was defined based on the OECD-adjusted household income (OECD, n.d.), and based on tertiles categorised as ‘low’, ‘intermediate’ and ‘high’. The Charlson Comorbidity Index (CCI) (Quan et al., 2011) (excluding breast and gynaecological cancer) was defined based on registration of the included diseases from hospital contacts in the NPR prior to the scheduled screening date, and the women were divided into a comorbidity score of ‘0’, ‘1’ and ‘≥2’.

### 2.4 Statistical analysis

The association between CCS coverage and BCS participation was estimated using generalised linear models with log link and the Bernoulli family regression models (Zou, 2004; Barros and Hirakata, 2003). We used prevalence rate ratios (PRR) with 95% confidence intervals (CI) and applied robust variance estimates to adjust for clustering of patients by general practice in both the unadjusted and adjusted models (Donner and K., 2000). The same analyses were applied to measure ANP and PNP in BCS as binary outcomes. Using stratified analyses, ANP were compared to participants in BCS (omitting PNP in these analyses), and subsequently PNP were compared to participants in BCS (omitting ANP in these analyses). All statistical analyses were conducted using STATA version 14.

### 2.5 Ethics

Since the study was based on data from registers only, no ethical approval was required according to Danish legislation and the National Committee on Health Research Ethics in the CDR (j. no. 181/2011). Approval for obtaining screening data was granted by the CDR's legal department and the Danish Data Protection Agency (j. no.: 1-16-02-109-09, j. no.: 1-16-02-376-16 and j. no.: 2009-41-3471).

## 3 Results

### 3.1 Participation

**Fig. 2:**
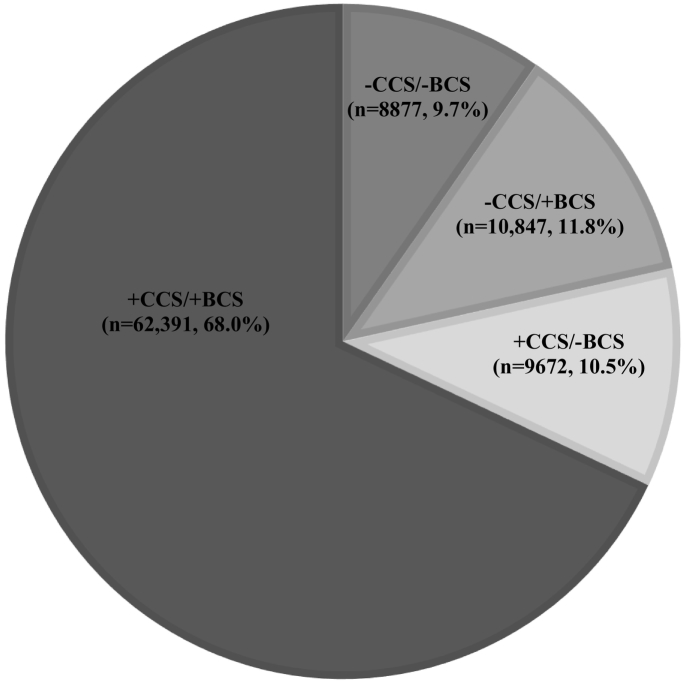
Distribution of cervical cancer screening (CCS) coverage and breast cancer screening (BCS) participation among 91,787 (100%) women invited to BCS in the Central Denmark Region (CDR) in 2008–2009.

Among the 91,787 women included, 21.5% were not covered by CCS and 20.2% were non-participants in BCS. In total, 68.0% (*n* = 62,391) were covered both by CCS and participated in BCS, and 9.7% (*n* = 8877) were neither covered by CCS nor participated in BCS (Fig. 2). Of the women covered by CCS, 13.4% (*n* = 9672) did not participate in BCS, and of the women who participated in BCS, 14.8% (*n* = 10,847) were not covered by CCS (Table 1).

**Table 1:** Distribution of cervical cancer screening (CCS) non-coverage, socio-demography and Charlson's comorbidity index (CCI) among participants (*n* = 73,238), non-participants (*n* = 18,549) and non-participants divided into active (ANP) (*n* = 8314) vs. passive (PNP) (*n* = 10,235) non-participants in breast cancer screening (BCS).

Variable	Participants in BCS^a^		Non-participants in BCS		*P*-value (chi^2^)	ANP in BCS		PNP in BCS		P-value (chi^2^)
	n	(%)	n	(%)		n	(%)	n	(%)	
All women	73,238	(79.8)	18,549	(20.2)		8314	(44.8)	10,235	(55.2)	
CCS coverage^b^					<0.001					<0.001
No smear	10,847	(55.0)	8877	(45.0)		3214	(36.2)	5663	(63.8)	
Smear	62,391	(86.6)	9672	(13.4)		5100	(52.7)	4572	(47.3)	
Age (years)					0.002					<0.001
50–54	25,691	(80.2)	6393	(19.8)		2567	(40.2)	3826	(59.9)	
55–59	25,988	(79.9)	6531	(20.1)		2879	(44.1)	3652	(55.9)	
60–64	21,289	(79.1)	5625	(20.9)		2868	(51.0)	2757	(49.0)	
Ethnicity					<0.001					<0.001
Danish/descendant	70,661	(80.5)	17,146	(19.5)		8001	(46.7)	9145	(53.3)	
Immigrant	2577	(64.8)	1403	(35.3)		313	(22.3)	1090	(77.7)	
Marital status					<0.001					<0.001
Married	52,761	(84.1)	9974	(15.9)		5061	(50.7)	4913	(49.3)	
Cohabiting	5500	(77.7)	1578	(22.3)		569	(36.1)	1009	(63.9)	
Living alone	14,977	(68.2)	6997	(31.8)		2684	(38.4)	4313	(61.6)	
OECD-adjusted household income					<0.001					<0.001
Low	14,870	(67.6)	7139	(32.4)		2568	(36.0)	4571	(64.0)	
Intermediate	26,677	(81.4)	6116	(18.7)		2857	(46.7)	3259	(53.3)	
High	31,691	(85.7)	5294	(14.3)		2889	(54.6)	2405	(45.4)	
Educational level					<0.001					<0.001
Low	22,396	(76.5)	6876	(23.5)		2676	(38.9)	4200	(61.1)	
Intermediate	31,714	(82.4)	6766	(17.6)		3314	(49.0)	3452	(51.0)	
High	18,354	(80.4)	4479	(19.6)		2206	(49.3)	2273	(50.8)	
Charlson Comorbidity Index					<0.001					<0.001
0	51,588	(82.1)	11,279	(17.9)		5099	(45.2)	6180	(54.8)	
1	6637	(77,2)	1961	(22.8)		781	(39.8)	1180	(60.2)	
≥2	3600	(71.2)	1455	(28.8)		656	(45.1)	799	(54.9)	

Numbers vary due to missing data.

a Defined as participation in the first round of breast cancer screening (BCS) in the Central Denmark Region (CDR) in 2008–2009.

b Defined as having a smear registered in the National Pathology Data Bank (NPDB) in the 5 1/2 years preceding the booking date for breast cancer screening (BCS).

### 3.2 Sociodemographic characteristics

A higher proportion of BCS non-participants was found among older women (60–64 years), immigrants, women living alone, women with low income, low educational level and a CCI ≥ 2 than among BCS participants (Table 1). A higher proportion of PNP was found among younger women (50–54 years), immigrants, non-married women, women with low income and low educational level, and women with a CCI of 1 (but not CCI ≥ 2) than among women who were ANP (Table 1).

### 3.3 Association between BCS participation and CCS coverage

The association between CCS coverage and BCS participation is presented in Table 2. In the adjusted analysis, women not covered by CCS were nearly three times more likely to be BCS non-participants than women covered by CCS (PRR_adjusted_ = 2.80, 95% CI: 2.68–2.93).

**Table 2:** The association (prevalence rate ratio (PRR)) between being covered by cervical cancer screening (CCS) and being non-participant in breast cancer screening (BCS), passive (PNP) or active (ANP) non-participant in BCS compared to being BCS participant.

	Non-participation in BCS^a^		PNP in BCS^b^		ANP in BCS^c^	
	Unadjusted PRR (95% CI)	Adjusted^d^ PRR (95% CI)	Unadjusted PRR (95% CI)	Adjusted^d^ PRR (95% CI)	Unadjusted PRR (95% CI)	Adjusted^d^ PRR (95% CI)
CCS coverage^e^						
Smear	1 (ref)	1 (ref)	1 (ref)	1 (ref)	1 (ref)	1 (ref)
No smear	**3.35** **(3.22–3.50)**	**2.80** **(2.68–2.93)**	**5.02** **(4.80–5.26)**	**3.99** **(3.80–4.19)**	**3.02** **(2.85–3.21)**	**2.50** **(2.34–2.68)**

Significant values are in bold" (as all results are significant, all estimates are in bold).

a Defined as non-participation in the first round of breast cancer screening (BCS) in the Central Denmark Region (CDR) in 2008–2009.

b Stratified analyses, comparing PNP to BCS participants omitting ANP's.

c Stratified analyses comparing ANP to BCS participants omitting PNP's.

d Adjusted for age, ethnicity, marital status, OECD-adjusted household income, educational level and Charlson comorbidity index score.

e Defined as having a smear registered in the National Pathology Data Bank (NPDB) in the 5 1/2 years preceding the booking date for breast cancer screening (BCS).

Women not covered by CCS were four times more likely to be PNP (PRR_adusted_ = 3.99 (95% CI: 3.80–4.19), whereas they were 2.5 times more likely to be ANP (PRR_adjusted_ = 2.50, 95% CI: 2.34–2.68) than women covered by CCS (Table 2).

## 4 Discussion

### 4.1 Main findings

This historical register-based cohort study found that CCS non-coverage was strongly associated with later BCS non-participation. Women who were not covered by CCS had a three-time larger likelihood of not participating in a later BCS than women covered by CCS. Of all women included in the study, one in ten was neither covered by CCS nor participated in BCS, and two thirds were both covered by CCS and participated in BCS.

### 4.2 Study limitations and strengths

A major strength of this study was that all information was retrieved from Danish registers, which are known to be valid and complete (Gjerstorff, 2011; Lynge et al., 2011; Bjerregaard and Larsen, 2011). This minimised information bias and ensured that we had accurate information on possible confounders at the level of the individual. Furthermore, since precise inclusion and restriction was possible, selection bias was minimised. Hence, the generalisability of the study is high, and the findings are thus applicable to other countries with similar screening programmes.

However, certain weaknesses should also be acknowledged. Firstly, women aged 60–64-year old were included in the CCS after 2007, yet the exact implementation date is not documented and differ across Denmark, thus it cannot be ruled out that some of the oldest women were not invited as intended to the CCS which may result in an underestimation of CCS coverage. However, this will not be associated with participation on BCS and will therefore not affect the association under study. Secondly, data on previous hysterectomy in the NPR and the NPDB were not available prior to 1977 and 1999, respectively (Lynge et al., 2011; Bjerregaard and Larsen, 2011). Thus, women with a hysterectomy or conisation who were 24 years or younger in 1977 were misclassified. Since hysterectomies are rare in young Danish women (Lam et al., 2015), this is of minor importance in the present study. Previous studies have found several factors that could be associated with both CCS and BCS screening participation but remain unadjusted for in this study, such as BMI (Cohen et al., 2008), urbanity (Leinonen et al., 2017; Jensen et al., 2012) and smoking (Harder et al., 2018; Lagerlund et al., 2015). Hence, residual confounding cannot be ruled out. Furthermore, this study does not indicate whether the link between CCS coverage and BCS participation is causal although this study, as one of the first in the fields, offers a temporal separation of CCS coverage following assessment of BCS participation. Future studies should investigate further in details the mechanism affecting non-participation across screening programmes.

### 4.3 Comparison with other studies

The results of this study are consistent with the results of previous international studies (Miedema and Tatemichi, 2003; Labeit and Peinemann, 2015; Augustson et al., 2003; Rakowski et al., 1995; Schoofs et al., 2017; Blackwell et al., 2008; Cummings et al., 2000; Sutton et al., 1994; Aro et al., 1999; Phillips et al., 1998), which also found an association between CCS and BCS participation, even though these previous studies were non-comparable in various ways. Different age groups were included and definitions of CCS and BCS participation varied among the studies from ‘ever being screened’ (Phillips et al., 1998) to ‘being screened within the previous year’ (Cummings et al., 2000). Moreover, previous studies were all mainly based on self-reported data and thus likely to be biased due to participation overestimation (Howard et al., 2009) and self-selection.

Screening behaviour is most likely determined by a complex range of persistent underlying factors, for example that the woman abstains from screening because she anticipates discomfort during examination and fears pain (Eaker et al., 2001; Aro et al., 2001; Waller et al., 2009) or embarrassment (Glasgow et al., 2000; Eaker et al., 2001; Aro et al., 2001; Waller et al., 2009). Women may also be afraid of the result of the screening (Glasgow et al., 2000; Aro et al., 2001; Waller et al., 2009) or may be convicted that it is better not to know (Glasgow et al., 2000; Aro et al., 2001). Other factors may be associated with absence of knowledge or simple misconceptions about cancer and screening, such as thinking that screening is unnecessary if there are no symptoms or believing that cancer will not strike (Glasgow et al., 2000; Eaker et al., 2001; Waller et al., 2009). Busyness may also be a barrier (Lagerlund et al., 2015; Eaker et al., 2001; Waller et al., 2009), and some simply do not attend for no specific reason (Waller et al., 2009). Moreover, the decision to participate in one type of screening may influence the decision to participate in another type of screening, causing a spill-over effect as suggested by Labeit et al. (Labeit and Peinemann, 2015). Finally, recent media focus on the negative side effects of screening, such as over-diagnosis and false positives/negatives, may also contribute to non-participation. In Denmark, focus has particularly been on BCS over-diagnosis, although the extent of overdiagnosis is heavily debated (Gøtzsche and Jorgensen, 2013; Njor et al., 2018). Thus, concern about over-diagnosis could explain why some women participate only in CCS, however, it is unlikely to explain the association between CCS and BCS participation.

The present study revealed that CCS non-coverage was more strongly associated with passive non-participation than with active non-participation in BCS. To our knowledge, two other studies (Jensen et al., 2012; Aro et al., 2001) have studied subgroups of non-participants in BCS, and none of these evaluated the relation to CCS coverage. To improve screening uptake, efforts should be targeted at PNP in particular because their decision may not have been informed and their participation could possibly be barred by factors that could be addressed in interventions. Thus, additional research is needed to further understand this association.

### 4.4 Implications

Non-participation in screening is an individual decision. However, research into reasons for non-participation and into how screening programmes may be improved is essential to ensure that access is truly equal and to achieve optimal societal effect of screening. Thus, the present study shows that women not covered by CCS are more likely to be BCS non-participants. This knowledge is important for GPs, who should be observant of women who do not participate in either CCS or BCS, since they are more likely also to be non-participants in the other screening programme. Furthermore, by addressing possible underlying factors related to non-participation, doctors may possibly be able to help address relevant participation barriers.

We showed that non-participation in CCS was more pronounced among PNP than among ANP in BCS. As noted above, these women may not have made an informed decision about non-participation. Thus, more information from doctors, health authorities and in the media could provide these women with information needed to make a decision about screening participation.

For women only participating in one screening offer, healthcare personnel may engage in conversation with the woman about participation in CCS when the woman attends BCS and vice versa. This conversation could also take place when a woman with no recent CCS participation calls to cancel her appointment for BCS.

Another possibility worth exploring is to introduce ‘integrated screening sites’ where women are invited to participate in more than one screening at the same time. This would likely increase participation (Chen et al., 2004). Women who do not participate in any of the screening programmes might also benefit from such an intervention since screening would become more accessible and less time-consuming. On the other hand, some women may find it overwhelming to attend two screenings on the same day. Furthermore, recommended screening intervals vary between CCS and BCS, which should also be taken into consideration.

## 5 Conclusion

This historical register-based cohort study shows that previous CCS behaviour was strongly associated with later BCS behaviour. However, the study also identified groups of women participating in only one type of screening. To raise participation, future interventions may target these groups, e.g. by establishing integrated screening sites.

## Abbreviations

ANP: active non-participants
ASR: age standardised incidence rate
BC: breast cancer
BCS: breast cancer screening
CC: cervical cancer
CCI: Charlson Comorbidity Index
CCS: cervical cancer screening
CDR: Central Denmark Region
CI: confidence interval
CPR: civil personal registration number
DCR: Danish Cancer Register
GP: general practitioner
ICD-10: International Classification of Disease, version 10
NPDB: National Pathology Data Bank
NPR: National Patient Register
PNP: passive non-participants
PRR: prevalence rate ratios
